# Nitrogen balance and gap of a high yield tropical soybean crop under irrigation

**DOI:** 10.3389/fpls.2023.1233772

**Published:** 2023-09-27

**Authors:** Leandro Moraes Zambon, Renan Caldas Umburanas, Felipe Schwerz, Jackellyne Bruna Sousa, Everton Servilho Teixeira Barbosa, Letícia Pacheco Inoue, Durval Dourado-Neto, Klaus Reichardt

**Affiliations:** ^1^Luiz de Queiroz College of Agriculture (ESALQ), University of São Paulo (USP), Piracicaba, Brazil; ^2^Agricultural Engineering Department, Federal University of Lavras (UFLA), Lavras, Brazil; ^3^Center of Nuclear Energy in Agriculture (CENA), University of São Paulo (USP), Piracicaba, Brazil

**Keywords:** Glycine max, biomass partitioning, N balance, soil inorganic N, plant nutrition

## Abstract

Nitrogen (N) is the most extracted and exported element by the soybean crop. In high yield tropical environments with irrigation, little is known about N accumulation in different soybean plant organs as well as the N balance. The objective of this study was to characterize soybean growth, N accumulation in plant organs, N balance, and N gap in a high yield tropical environment. This study was performed in a homogeneous field, in a soil with low organic matter, with 20 kg ha^-1^ of N, under furrow fertilization. Evaluations were performed ten times, temporally distributed from emergence to senescence. The soybean cultivar used was ‘RK7518 IPRO’ and was sown with row spacing of 0.45 m and a seeding rate of 300,000 plants ha^-1^. Plant N partition, N from the biological N fixation (BNF), grain yield, crop harvest index (HI), N harvest index (NHI) with and without root contribution were evaluated. Also, at the grain filling stage the N gap was evaluated from the soil by difference between whole plant accumulated N and the amount of N from BNF. The average grain yield was 6,470 kg ha^-1^ and leads to a negative partial balance of N of -33.4 and -42.8 kg_[N]_ ha^-1^ with and without roots, respectively. The N gap from the soil was 231.7 kg_[N]_ ha^-1^. It is recommended to adopt techniques that increase the efficiency of BNF and the soil N accumulation to balance these production systems in the medium to long term.

## Introduction

The soybean [*Glycine max* (L.) Merr.] crop is one of the largest vegetable protein and oil sources, with about 40% protein in the grain, mainly used in animal feed (Ciampitti and Salvagiotti, 2018). In addition, soybeans present all the essential amino acids for humans and are of low cost when compared to other protein sources (Carrera et al., 2011). The increase in world population has increased the demand for food, and Brazil is one of the countries with the greatest potential to atend this demand. In 2020, and worldwide, approximately 127 million hectares were cultivated with soybeans, which produced 353 Mg of grains (Food and Agriculture Organization (FAO) of the United Nations, 2023).

The average yield of Brazilian soybean crops in the 2020/2021 season was 3,525 kg ha^-1^ (Companhia Nacional de Abastecimento, 2023), however, yields above 5.0 Mg ha^-1^ are frequently achieved in high yield tropical environment under irrigation. Nitrogen (N) is the element mostly extracted and exported by the soybean crop, and with an average content of 6.5% N in the seeds, this element is key in the search for high yields and seed quality (Ciampitti and Salvagiotti, 2018). To produce 1.0 Mg_[grains]_, the soybean crop extracts approximately 80 kg_[N]_ by the aerial part of the plants (Salvagiotti et al., 2008). Thus, for yields around 6.0-8.0 Mg ha^-1^, this amounts to about 480-640 kg_[N]_ ha^-1^ (La Menza et al., 2017). Studies have shown that soybean producers who performed fertilization with N had their yield increased. Several authors described yields of 4 Mg ha^−1^ or more, due to mineral N application (Brevedan et al., 1978; Wesley et al., 1998; Mendes et al., 2008). According to Ortez et al. (2018), in a recent study with several genotypes, the authors observed gains of 12% and 4% in the soybean yield in the USA and Argentina, respectively, using higher rates of N (670 kg ha^−1^) applied during the reproductive growth stage when compared to the control without N, which demonstrates that soybean can respond to complementary N. However, N fertilization has been considered a controversial topic in the literature. There are researches showing that N fertilization resulted in same yields as the control (without mineral N) (Ham and Caldwell, 1978; Schmitt et al., 2001). According to Saturno et al. (2017), soybean producers did not have increase in the yield with fertilization of 200 kg ha^−1^ of N.

The extraction and accumulation of N by soybeans depend in general on two sources: the biological nitrogen fixation (BNF) and the absorption of mineral soil N. The relative contribution of each source is the result of environmental conditions, agronomic management practices and genetic factors (Santachiara et al., 2017). For environments with yield potential above 5,000 kg ha^-1^, information related to the relative contribution of these two sources is scarce (Salvagiotti et al., 2008). In addition, little is known about the capacity of BNF to supply the N demand of plants in high yield environments, as well as about the N balance in the environment. Most of the studies involving BNF have focused only on the determination of the N_2_ fixed in the aerial part, disregarding the contribution of the roots, which must also be considered to better understand the contribution of BNF, as well as its impact on N balance (Ciampitti and Salvagiotti, 2018). In soybeans, a dilution effect on protein content, evaluated through nitrogen content, has been reported due to increased grain yield in temperate (La Menza et al., 2017), subtropical (Umburanas et al., 2022; Makuch et al., 2023) and tropical environments (Pierozan Junior et al., 2020).

Most studies that analyze the extraction of N by soybean plants, as well as the contribution of BNF, do not present the partition of the contribution to their respective organs. In other words, there is need to quantify the accumulation of nitrogen and subsequent translocation of the element to the seeds in formation, using cultivars adapted to intensive production systems (Bortolon et al., 2018). Therefore, to ensure future gains in soybean seed yield, it is necessary to understand the processes associated with the absorption and assimilation of N, as well as understand the contribution of BNF and redistribution of N to seeds (Mastrodomenico and Purcell, 2012; Santachiara et al., 2017).

In this study, we consider partial N balances as accounting for all input and output of N in the system (Landriscini et al., 2018), and the N gap represents the gap of N left in the system as a function of the amount of N exported at harvest by grains, here with a negative value. Studies performed quantifying N uptake and balance in soybean plants were carried out mostly in temperate environments (La Menza et al., 2017; Landriscini et al., 2018). However, such information does not cover tropical environments. Therefore, it is important to study the N balance in tropical environments, mainly due to the contrasting climatic conditions, such as higher air temperature and greater availability of solar radiation as compared to temperate environments (Alvares et al., 2013). In this context, the following hypothesis was studied: Soybean production in a tropical environment mostly generates a negative Nitrogen balance in the production system due to the amount of exported N.

Partial balances of N require an adequate estimate of the BNF, and they are useful to estimate the magnitude of the loss or gain of N in the crop system (Landriscini et al., 2018). Negative values indicate that the amount of N exported at harvest by grains is greater than the N fixed by the crop, resulting an N depletion in the soil (Ciampitti and Salvagiotti, 2018) which can have negative long-term consequences.

This study contributes with relevant information according to the following objectives: i) characterization of the temporal variation of N accumulation in soybeans by quantifying the growth, development and leaf area index; ii) determining the N content and accumulation in the different parts of the plants as well as the remobilization of the element to seeds in formation; iii) calculating the rates of dry matter accumulation and N extraction during the crop cycle; iv) determining the content of nitrate and ureide in the soybean stem and estimating the relative contribution of biological nitrogen fixation; v) calculating the grain and the N harvest indexes; and vi) evaluating the N balance in the system considering soybean roots or not, and making the N gap of the grown crop.

## Materials and methods

### Site and experimental design

The study was carried out in Piracicaba, SP, Brazil (22°41’S, 47°38’W, 546 m) in a clayey Oxisol, during the crop cycle 2017/2018. The climate of the region is Cwa (Köppen), with rainy summer and winter drought. The previous crops grown on the field were maize (*Zea mays*) and millet (*Pennisetum glaucum*). Physical and chemical soil attributes are shown in **Table 1** for the 0–0.2 and 0.2–0.4 m surface soil layers.

**Table 1:** Soil characteristics of the experiment site.

Depth	pH	O.M.	K^+^	Ca^2+^	Mg^2+^	H+Al	Al^3+^	CEC	P	SO_4_-S	B	Fe	Mn	Zn	Cu	Particle size (mm)	g kg^-1^
m	CaCl_2_	g dm^-3^	——–cmol_c_ dm^-3^ ——–						mg dm^-3^		—mg dm^-3^—					Clay (<0.002):	514
0-0.2	5.7	9	0.5	3.6	1.7	2.8	0	8.6	30	7	0.4	12	11	2	4	Silt (0.053 -0.002):	146
0.2-0.4	5.5	7	0.3	3.3	1.3	2.8	0	7.7	11	25	0.3	9	65	1	3	Sand (0.053-2):	340

CEC, Cation exchange capacity; O.M., Organic matter was determined by the Walkley-Black method; P, K^+^, Ca^2+^, and Mg^2+^ were extracted by ion exchange resin; Al^3+^ was extracted by KCl 1 mol L^-1^; H+Al was extracted by SMP method; B was determined by hot water; and Fe, Mn, Zn, and Cu were determined by DTPA.

The trials consisted of a randomized complete block design with six replicates. The experimental area was divided into 60 plots. Each plot of 2.25 × 4.0 m contained 5 rows, 4 m long and 0.45 m row spacing. The useful sampling area was limited to the three central rows, excluding 0.5 m from the edges, resulting in 3.75 m^2^ for plant sampling. Plant evaluations were performed only once in each plot, avoiding problems with experimental error and border effect. During the complete growing cycle samplings were performed in six randomly chosen plot every 14 days, totalizing 9 plant sampling dates plus harvest during the soybean cycle. The chosen soybean cultivar was ‘RK7518 IPRO’, with indeterminate growth habit, of medium cycle, from the maturity group 7.5. Sowing was carried out on October 10, 2017, five row seeder with 0.45 m row spacing were used and sowing density was 30 seeds per m^-2^. Seeds were treated with Fipronil (250 g L^-1^), Pyraclostrobin (25 g L^-1^) and Thiophanate-methyl (225 g L^-1^) at a rate of the formulated 2.0 mL kg^-1^ of seeds; inoculated with strains of *Bradyrhizobium japonicum* (SEMIA 5079) and *B. elkanii* (SEMIA 5019) at a concentration of 5×10^9^ cells mL^-1^. Furrow fertilizer application at sowing consisted of 20 kg ha^−1^ of N, 70 kg ha^−1^ of P_2_O_5_ (triple superphosphate) and 40 kg ha^−1^ K_2_O (potassium chloride). Weeds, pests and diseases were adequately controlled according recommended management practices (EMBRAPA, 2020).

In each evaluation plants in 0.36 m^2^ per plot were collected. During each sampling, in the same area, root samplings were carried out in a soil monolith with dimensions of 20 × 30 × 25 cm, which was sieved for root biomass evaluation. Ten samplings were made during the crop cycle, at the phenological stages V_E_, V_4_, V_8_, R_1_, R_2_, R_4_, R_5.2_, R_6_, R_8_ (Fehr and Caviness, 1977) and harvest. The collected plants were separated into root, stem, leaf and pod (when present). The petioles were grouped together with the stem.

### Evaluations

Roots were washed, and the leaf area was evaluated through a leaf area integrator (model LI-COR LI-3000, LI-COR, Lincoln, NB, USA). The leaf area index (LAI) was calculated for the stages V_4_, V_8_, R_1_, R_2_, R_4_, R_5.2_ and R_6_. Samples were dried in a forced airflow oven at 60°C for 72 hours and ground (0.1 mm) for quantification of nitrate (N-NO_3_^-^) by the method proposed by Cataldo et al. (1975), expressing data in mM [N-NO_3_^-^] g^-1^ [DM]. The ureide content (N-Ureides) in mM [N-Ureides] g^-1^ [DM] was determined as proposed by Young and Conway (1942). Atmospheric derived nitrogen (ADN) from BNF (%) was calculated by the ratio: [N-Ureide/(N-ureide + N-NO_3_^-^)], as proposed by Herridge (1982). For total N (TN) analysis by dry combustion (Keeney and Bremner, 1967), only samples of stages V_8_, R_2_, R_4_ and R_6_ were used. With the obtained data, a polynomial model was generated to estimate the missing TN data.

Assuming 16% N content in the protein, the N accumulation in the plant organs was estimated. The remobilization of N from stems and roots was estimated by the difference in the accumulation of N in the organs between R_5.2_ and R_8_ (Rotundo et al., 2014). To better understand the contribution of BNF to grain production, a final source balance of N was made subtracting the maximum plant assimilation of N by BNF from the total N exported by grains in kg ha^-1^. The N gap was calculated by subtracting the total N accumulation in the plant in R_6_ from the total ADN (Ciampitti and Salvagiotti, 2018). The apparent N harvest index was calculated by the relationship between the amount of N exported by the grains at harvest and the total accumulation of N at R_8_ in the aerial part of the plants (Bender et al., 2015) considering or not the N accumulated in the root system.

At final harvest (R_8_) to evaluate grain yield, the plants present in 3.6 m^2^ were evaluated. Harvest was performed on March 2, 2018. Grain yield was determined, and the value was corrected to 13% moisture. Two harvest indexes were calculated through the relation between the total mass of dry grains and the total mass of the aerial part at R_8_, including roots or not.

### Data adjustment

Data of DM and N accumulation were adjusted to a sigmoidal model using the Table Curve 2D software. Data of NO_3_^-^, ureide and ADN were submitted to variance analysis, using the Tukey test at 95% significance, with the software R v.3.5.0. The chosen sigmoidal model is represented by Eq. 1:


(1) $$\text{Y=}\frac{\text{a}}{{1+e}^{-\frac{\left(\text{t-b}\right)}{\text{c}}}}$$

where Y can be either DM or N accumulation, t the time in days after emergence DAE and a, b and c are empirical parameters obtained from the Table Curve software by minimizing deviations. This model allows the calculations of growth rates (dY/dt, kg ha^-1^ d^-1^). The maximum growth rate is given by a/4c, the moment of the maximum growth rate is b and the $\underset{t\to \infty }{\lim}Y=a.$ The first derivative dDM/dt (kg ha^-1^ d^-1^) of the sigmoidal model of DM leads to a bell-shaped curve [Eq. 2], indicating that the rate attains to a maximum:


(2) $$\frac{\text{dDM}}{\text{dt}}=\frac{\text{a}{\text{.e}}^{-\frac{\left(\text{t-b}\right)}{\text{c}}}}{\text{c}.{\left[{1+e}^{-\frac{\left(\text{t-b}\right)}{\text{c}}}\right]}^{2}}$$

The maximum value of dDM/dt is obtained through the second derivative according to Eq. 3:


(3) $$\frac{{\text{d}}^{2}\text{DM}}{{\text{dt}}^{2}}=\frac{\text{a}{\text{.e}}^{-\frac{\left(\text{t-b}\right)}{\text{c}}}.\left\{2{\text{.e}}^{-\frac{\left(\text{t-b}\right)}{\text{c}}}-\left[{1+e}^{-\frac{\left(\text{t-b}\right)}{\text{c}}}\right]\right\}}{{\text{c}}^{2}.{\left[{1+e}^{-\frac{\left(\text{t-b}\right)}{\text{c}}}\right]}^{3}}$$

Making d^2^DM/dt^2 = ^0 we verify that the maximum rate occurs at:


(4) t=b

and the maximum rate (dDM/dt) max at t = b, being:


(5) $$\frac{\text{dDM}}{\text{dt}}\left(\frac{{\text{d}}^{2}\text{Y}}{{\text{dt}}^{2}}=0\right)=\frac{\text{a}}{4\text{.c}}$$

The value of DM at the inflection point (t = b) is given by Eq. 6:


(6) $$\text{DM}\left(\frac{{\text{d}}^{2}\text{Y}}{{\text{dt}}^{2}}=0\right)=\frac{\text{a}}{2}$$

The expected maximum dry mass (DMmax) at maturity tends to:


(7) DMmax =a

### Meteorological data

**Figure 1:**
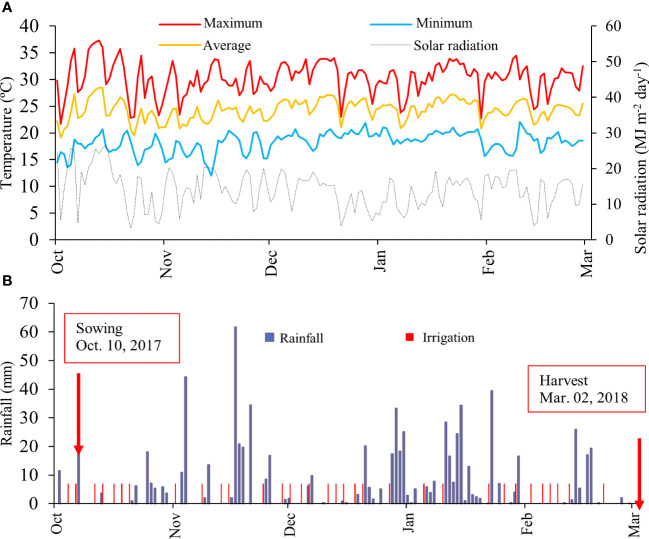
Daily total solar radiation, daily maximum (red), average (orange) and minimum (light blue) air temperature **(A)**; and rainfall and irrigation **(B)** across the growing season.

The solar radiation (MJ m^-2^ d^-1^) was obtained from a portable meteorological station installed at the experimental site (**Figure 1A**). Temperature and rainfall data were collected by a meteorological station located approximately 1 km from the experiment. During dry spells, the crop was irrigated every other day with 7 mm to assure an optimum water regime (**Figure 1B**).

The meteorological data presented in **Figure 1** demonstrate that the conditions of air temperature and solar radiation were adequate throughout the crop cycle. Rainfall was not sufficient to meet the water demand of the crop. In this case, it was necessary to carry out 33 irrigation events during the cycle, therefore, 231 mm were added through irrigation. In this context, all meteorological conditions were favourable during the evaluated production cycle.

### Data analysis

A descriptive analysis of the data was performed using the Excel platform, with the objective of characterizing and defining the mean values and standard deviation of the sample according the results obtained. Also, the equations of the dry matter accumulation and N accumulation models were performed, with the statistical data from the models.

## Results

**Figure 2:**
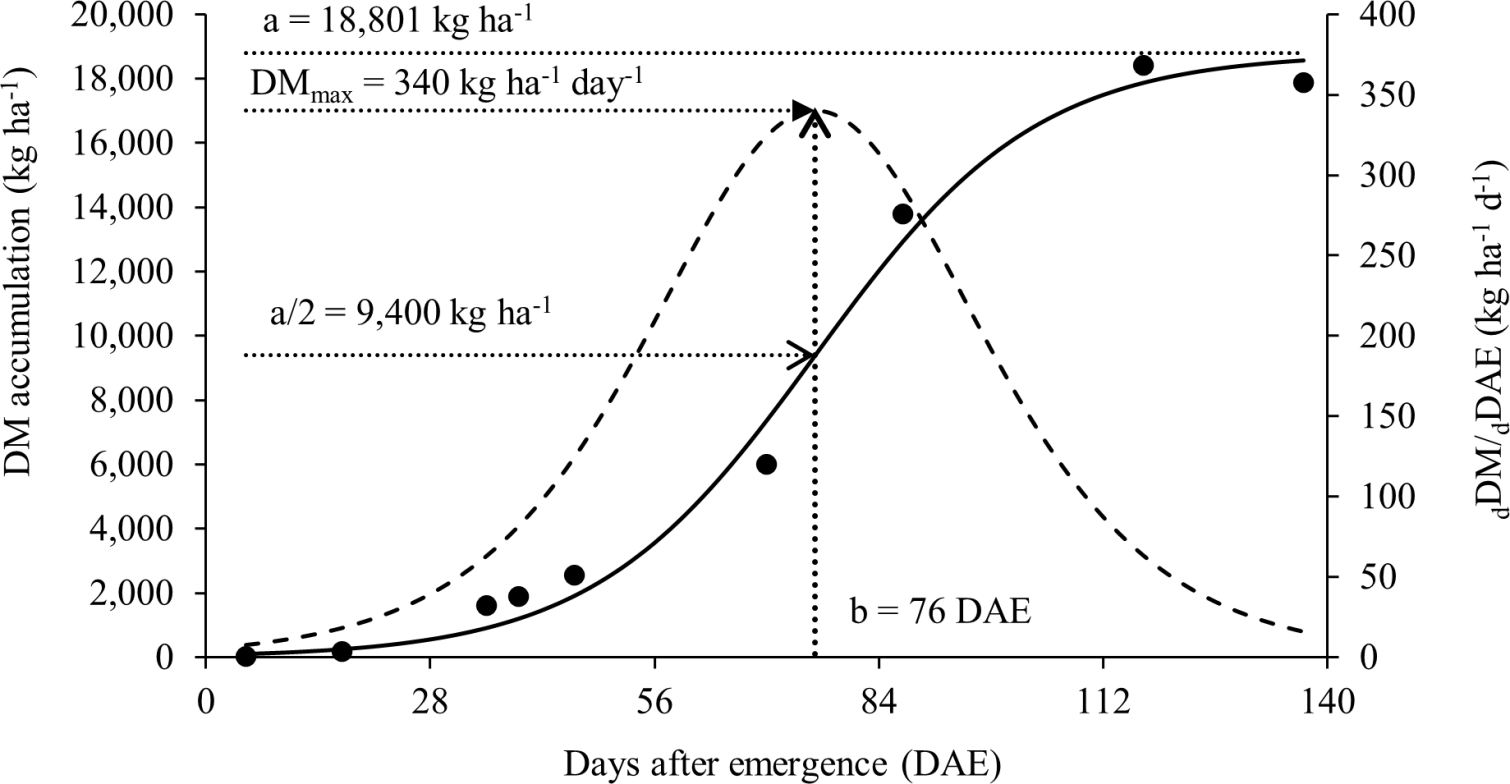
Soybean total dry matter (DM) accumulation as a function of time (days after emergence, DAE) (points are experimental and solid line follows equation (2)). DM accumulation rate dDM/dDAE (bell shaped dotted line). DMmax is the maximum accumulation rate, parameter ‘a’ the limit of total DM accumulation and parameter b the moment DAE of the maximum accumulation rate.

The results presented in this study can help soybean producers to understand how nitrogen dynamics and partitioning work in the plant and how this can affect soybean growth and yield. In this context, the total dry matter accumulation per plant adjusted to the sigmoidal model can be seen in **Figure 2**. This study had a maximum total dry matter accumulation rate of 340 kg ha^-1^ day^-1^ during the reproductive phase and a total accumulation of DM in the aerial part of 18,398 kg ha^-1^ at R_8_, in addition to a high rate of N accumulation. The statistical data and parameters from the model are presented in **Table 2**.

**Table 2:** Statistical data from the models: dry matter (DM) accumulation (**Figure 2**) and N accumulation (**Figure 3**).

DM accumulation						
Parameter	Value	E	t	Confidence interval		P>|t|
				Minimum	Maximum	
a	18,801.6	801.6	23.5	16,840.1	20,763.1	<.0001
b	76.1	2.5	29.9	69.8	82.3	<.0001
c	13.8	2.0	6.9	8.9	18.7	0.0005
N accumulation						
Parameter	Value	E	t	Confidence interval		P>|t|
				Minimum	Maximum	
a	631.1	31.0	20.4	555.4	706.9	<.0001
b	84.0	3.0	27.9	76.7	91.4	<.0001
c	15.9	2.2	7.2	10.5	21.2	0.0004

R^2^ = 0.991; R^2^_ADJ_ = 0.986; F = 361.2.

**Figure 3:**
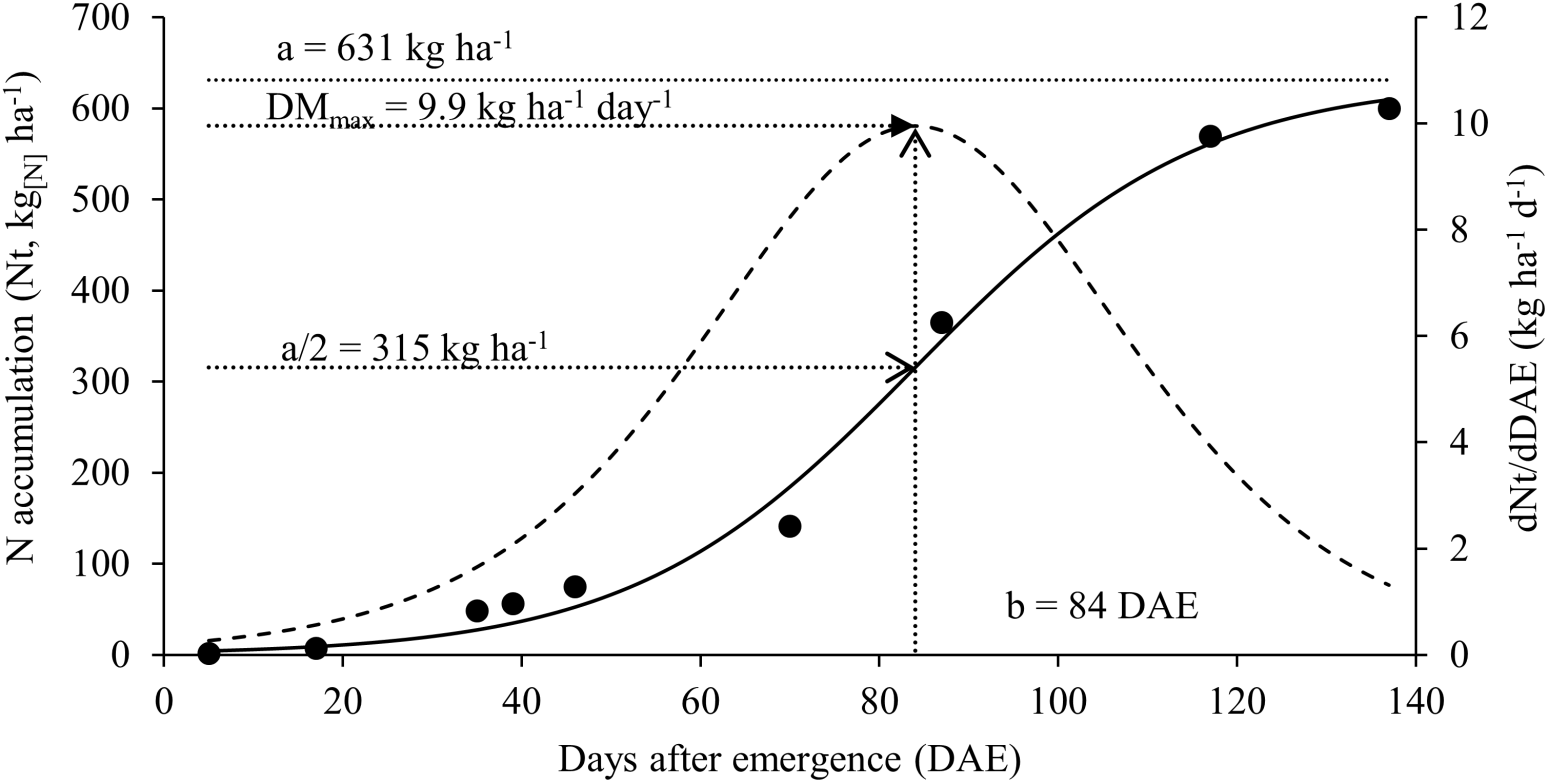
Nitrogen accumulation [continuous solid line] and nitrogen accumulation rate [dashed line] for soybean plants.

**Figure 4:**
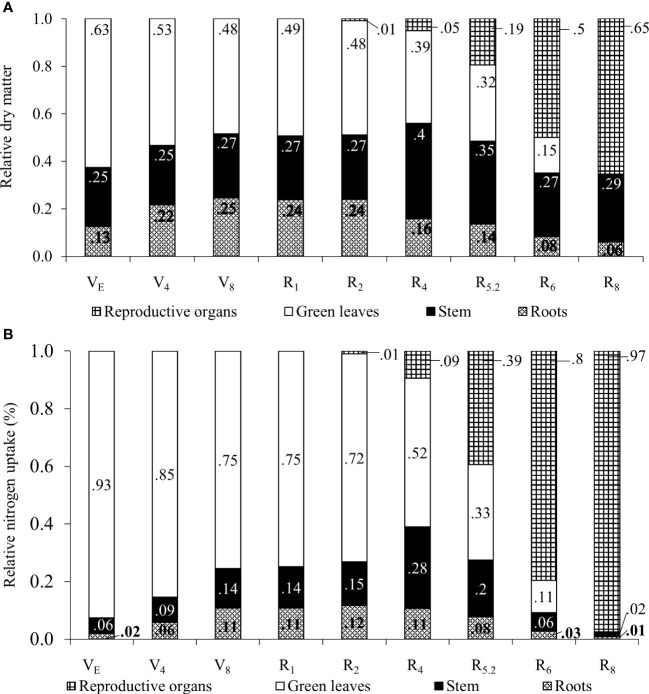
Relative proportion of dry matter **(A)** and nitrogen accumulation **(B)** in plant organs for soybean plants.

The relative dry mass of the organs shown in **Figure 4A**, also indicates translocations to the reproductive organs. Up to the R_2_ stage soybean plants keep the higher relative proportion of N in leaves while root partition is maintained.

**Figure 5:**
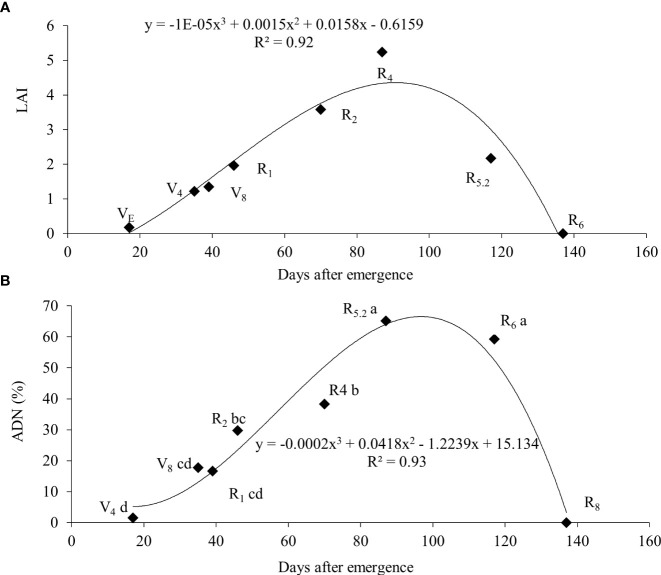
Leaf area index (LAI) **(A)** and atmospheric-derived nitrogen (ADN) **(B)** throughout the cycle for soybean plants.

From R_4_ onwards, the relative participation of leaves in the total plant mass decreased as a result of an increase in the mass of stems and roots, as well as the appearance of the first pods. At R_6_, only 15% of the total mass of the plant was made up of leaves, this fact being attributed to fall due to senescence and mass increase of the pods. The soybean leaf area index increased almost linearly from emergence (VE) until grain filling (R_5.2_), with a subsequent decline in R_6_ and the absence of leaf area in R_8_ (**Figure 5A**).

The total and fractional average dry matter dynamics and N concentration of soybean over the phenological stages are presented in **Table 3**. The concentration of N in the leaves was higher in the first phenological stages with a reduction over the crop cycle. This evidences the higher N concentration in the initial stages and a dilution in the final stages, since even with a lower concentration of N at R_5.2_, maximum plant N accumulation occurred in this phase (**Table 3**). Although an increase in the concentration of N was expected in the pods from R_6_ to R_8_ due to grain filling and the remobilization of N, the behavior of the concentration of N in the pods can be explained by a possible dilution effect, since there was a significant increase in mass in this period, which generated maximum N accumulation in the pods at R_8_. In the roots, the maximum accumulation was found in R_5.2_ with 28.2 kg_[N]_ ha^-1^, being associated with the maximum root mass determined at this stage and with N content of 15.1 g kg^-1^.

**Table 3:** Dry matter, nitrogen concentration and total nitrogen accumulation for the plant organs of soybean at the different phenological stages.

Organ	Phenological stages									HI (%)
	V_E_	V_4_	V_8_	R_1_	R_2_	R_4_	R_5.2_	R_6_	R_8_	
	Dry matter (DM) (kg ha^-1^)									
Root	4.3	40	395	448	616	945	1,866	1,524	1,076	
Stem	8.7	47	435	501	697	2,413	4,820	4,944	5,115	
Leaf	22	100	777	923.7	1,231	2,335	4,441	2,755	0	
Reproductive organs (RO)	0	0	0	6.5	20	295	2,671	9,174	11,693	
Grains									5,629	
Total	35	187	1607	1,878	2,563	5,988	13,798	18,397	17,884	31. 5
Total of aerial part (AP)	30	146	1212	1,430	1,947	5,043	11,932	16,873	16,808	33.5
Relative (%) DM in roots	13	21	25	24	24	16	14	8	6	
Relative (%) DM in RO	0	0	0	0	1	5	19	50	65	
Relative (%) DM in AP	87	78	75	76	76	84	86	92	94	
	Total nitrogen concentration (g kg^-1^)									
	V_E_	V_4_	V_8_	R_1_	R_2_	R_4_	R_5.2_	R_6_	R_8_	
Roots	6.7	9.6	13	14	14	16	15	11	5.0	
Stem	9.6	13	15	16	16	17	15	7.4	1.9	
Leaves	64	57	47	45	44	31	27	23	–	
Reproductive organs					37	45	54	49	50	
Grains									66	
	Total nitrogen accumulation (kg ha^-1^)									NHI (%)
	V_E_	V_4_	V_8_	R_1_	R_2_	R_4_	R_5.2_	R_6_	R_8_	
Roots	0.03	0.4	5.2	6.0	8.7	15	28	16	5.4	
Stem	0.08	0.6	6.6	8.0	11	40	72	37	9.7	
Leaves	1.4	5.7	36	42	54	73	121	64	0	
Reproductive organs (RO)	0	0	0	0	0.7	13	144	453	585	
Total	1.5	6.7	48	56	74	141	365	569	600	61.8
Total of aerial part (AP)	1.4	6.3	43	50	66	126	337	553	594	62.4
Relative (%) N in roots	2	6	11	11	12	11	8	3	1	
Relative (%) N in RO	0	0	0	0	1	9	39	80	98	
Relative (%) N in AP	99	94	89	89	89	89	92	97	99	

HI, harvest index; NHI: nitrogen harvest index; Reproductive organs refers to the flower in R_1_ and R_2_ and the pod in the other phenological stages; V: vegetative stage; R: reproductive stage; Phenological stage according to Fehr and Caviness (1977).

At R_6_ and R_8_ the storage of root N reduced to 16 and 5.4 kg ha^-1^ respectively, this drop is attributed to the remobilization of N to the pods, in the order of 66.8%, and due to dead roots. Regarding stems, 71.8 kg ha^-1^ was the maximum value determined at R_5.2_. At R_8_, only 9.7 kg_[N]_ ha^-1^ were found in this organ, suggesting 87.2% remobilization. Also, at R_5.2_, maximum N accumulation occurred for the leaves (121.2 kg ha^-1^). Although the N content was relatively low compared to previous stages of development, the maximum leaf mass was found at R_5.2_, which contributed to this result. As leaves fallen on the soil due to senescence were not collected, the remobilization of N by the leaves could not be calculated. For pods, a gradual increase in N accumulation was observed up to R_8_ (584.7 kg ha^-1^). Through the relation between the maximum accumulation of N in the aboveground part and yield, the soybeans extracted 91.87 kg_[N]_ ha^-1^ for each Megagram of harvested seeds.

Although not accounted for in our study, part of the N contained in the dead leaves was certainly remobilized to the seeds before fall. The greater N remobilization from the stem may be associated with the high demand for N by the seeds, as well as the time interval between R_6_ and R_8_ in which the plants had a long period to remobilize N.

The maximum accumulation of N found in vegetative organs at stage R_5.2_ is related to the maximum accumulation of biomass that occurred between the period from R_4_ to R_5.2_ as well as the maximum rate of N accumulation that also occurred in this interval (**Figure 3**). At the beginning of the crop cycle, N accumulation rates were low and gradually increased until reaching the maximum at 84 DAE (range between R_4_ and R_5.2_) and after that, the rate of N accumulation drops again as plants move towards maturation.

The N content determined in the seeds was 65.9 g kg^-1^, which suggested a protein content of 41.18%. It can be observed that between the V_E_ and R_2_ stages, ¾ or more of the N content was stored in the leaves (**Figure 4B**). From R_4_ onwards, the relative proportion of N stored in the leaves decreased with the appearance of pods in the plant. In stems, the maximum relative N accumulation occurred in R_4_ (28%) and from this point on, the ratio reduces to 2% of the N accumulated in the plant at R_8_, certainly due to the remobilization of the element to the pods. Regarding pods, an increase in the relative proportion of N was found from R_4_ (9%) to R_8_ (97%). The remobilization of N from vegetative organs, as well as the absence of leaves at this stage, contributed to this result.

Although our root sampling methodology has not accounted for all roots present in the soil, great effort was made to collect the maximum amount. Thus, the result presented here can infer the contribution of root N in the system and contribute to future calculations of the N balance in the system. The yield on a dry basis (5.6 Mg ha^-1^) lead to a total export of 371 kg_[N]_ ha^-1^. The maximum accumulated N from BNF occurred at R_6_ with 328.2 kg_[N]_ ha^-1^ in aboveground and 9 kg ha^-1^ contributed by the roots. These numbers show that the partial balance of N was negative (total export – BNF), -33.4 and -42.8 kg_[N]_ ha^-1^ with and without roots, respectively. Subtracting the total N accumulated by the whole plant (considering the roots) at R_6_, 569 kg_[N]_ ha^-1^, by the N coming from the BNF, results in a N gap of 231.7 kg_[N]_ ha^-1^.

The greatest nitrate concentration in the stem was at V_4_ (15.9 mM g^-1^) and wit a gradually reduction up to R_6_. In contrast, the ureide content was low (0.24 mM g^-1^) at V_4_ and had an increase up to R_5.2_ a subsequent reduction at R_6_ (**Table 4**). An increase in BNF activity was observed from the vegetative stages until reaching maximum efficiency at R_5.2_ (65.2%), with a subsequent decline to 59.3% at R_6_ (**Figure 5B**).

**Table 4:** Nitrate (NO_3_^-^) and ureide content expressed in mM g^-1^ of dry stem during the phenological stages.

Phenological stage	Nitrate	Ureid
	mmol g^-1^	
V_4_	15.9 ± 3.04	0.24 ± 0.07
V_8_	5.63 ± 1.24	1.22 ± 0.24
R_1_	7.36 ± 1.12	1.47 ± 0.10
R_2_	3.78 ± 0.94	1.60 ± 0.57
R_4_	3.48 ± 0.82	2.16 ± 0.27
R_5_._2_	2.20 ± 0.83	4.13 ± 0.55
R_6_	1.74 ± 0.59	2.53 ± 0.53
CV (%)	29	25

± means average deviation; V: vegetative stage; R: reproductive stage; Phenological stage according to Fehr and Caviness (1977).

## Discussion

The soybean grain yield was 5.6 Mg ha^-1^ (dry basis) or 6.5 Mg ha^-1^ (13% moisture) and generated a harvest index of 33.5% or 31.5% when the root mass was accounted for, which corroborates with Salvagiotti et al. (2009). However, values between 38.1% and 48.8% were reported by Roekel and Purcell (2014). Existing variations in HI may be related to the irrigation, environment, cultivar used, growth habit, climatic conditions during the cycle, among other factors. Our result may be related to factors that favored the vegetative growth, such as adequate planting time, levels of nutrients, water availability and satisfactory control of biotic factors. In this context, the higher stem mass of plants observed in this study in R_8_ may have generated a certain reduction in the harvest index.

The higher relative proportion of leaves observed up to the R_2_ stage may be a strategy of the plant in supplying the photoassimilate products to roots (Portes and Araújo, 2012). At R_6_ the senescence and mass increase of the pods decrease leaf partition to 15% or lower, as evidenced in our results and corroborated by Zajac et al. (2017) and Gaspar et al. (2017). Isoda et al. (2010) reported LAI values close to 8 for a yield of 9,200 kg ha^-1^. A higher LAI ensures that the plants remain for a longer time in a condition of maximum light interception (Tagliapietra et al., 2018). These authors concluded that the optimum LAI at R_5_ to reach the potential yield in cultivars of indeterminate habit should be around 6. We may suggest that the soybeans grown in this experiment produced close to the potential yield since the maximum measured LAI was 5.23 and there were no limitations in water and nutrients, as well as a satisfactory control of biotic stresses.

The higher concentration of nitrate in vegetative stages (mainly V_4_) may be related to the N fertilization that was carried out at sowing, as well as to the low activity of BNF and the higher OM content of the upper soil layers, in the same pattern as reported by Córdova et al. (2019). Thus, as the root system developed in depth, the availability of NO_3_^-^ probably decreased and the BNF process was intensified, thus explaining the relationship of these two forms of N in relation to the phenological stages. After R_2_, nitrate reductase activity decreased rapidly and nitrogenase increased, which evidences an inverse relationship in the behavior of these two enzymes during plant development. As the plants developed new leaves, the nutrition of the microorganisms benefited and the N_2_ fixation process intensified.

Some management practices can affect BNF. It was reported that the application of amino acids such as glutamate, phenylalanine, cysteine and glycine in early soybean development stages increased the ureide content, which infer that there was greater BNF by the plants, due to the signaling role promoted by the amino acids (Teixeira et al., 2018). Also, the form of pre-inoculation of the seed with *Bradyrhizobium* sp. before sowing affects BNF performance. Inoculation should be carried out as close to sowing as possible, since the longer the time lapse between inoculation and sowing, the lower the BNF performance will be (Sartori et al., 2023).

Concentrations of N in the roots of 4.9, 7.6 and 4.5 g kg^-1^ were determined for the phenological stages V_2_, R_1_ and R_8_, respectively, by Pal and Saxena (1976). In stems, levels of 15.1, 9.9 and 4.5 g kg^-1^ were found at stages R_3_, R_6_ and R_8_, respectively, by Zajac et al. (2017). To sustain the high demand for N for seeds, soybeans accumulate this element in the vegetative parts and remobilize it to grains during the grain filling phase (Gaspar et al., 2017).

Average values between 66-69% of N remobilization after R_5.5_ were found by Gaspar et al. (2017), and Bender et al. (2015) found 65% of remobilization to leaves and 32% for stems. It is therefore evident that approximately 1/3 of the total N accumulated in the leaves is not remobilized.

In another study with the same cultivar used in our study, whose yield varied between 2,500 and 6,000 kg ha^-1^, protein content levels of 41% and 38% were determined (La Menza et al., 2017; Umburanas et al., 2018; Makuch et al., 2023). In our study, even with a yield greater than 6,000 kg ha^-1^, plants were able to maintain a higher protein content. This fact can be attributed to the irrigation, the high accumulation of N found in the vegetative parts of the plants at R_5.2_ that was redistributed to the seeds in formation, as well as to the continuity of N extraction by the plants, mainly through the BNF process. The most productive modern cultivars demonstrated not only a greater capacity for N remobilization but also a high ability to extract and accumulate N after R_5_ (Gaspar et al., 2017). In addition, the availability of N in the reproductive period determines the seed yield directly by increasing the supply of N for seed growth (Kinugasa et al., 2012).

The greater accumulation of N and the relative proportion in the leaves has a physiological purpose. The N present in the leaves of plants occurs mainly in the form of the enzyme ribulose-1,5-bisphosphate carboxylase/oxygenase (RuBisCO), responsible for the assimilation of atmospheric CO_2_, and normally, a great relationship between N per unit leaf area and photosynthesis are reported (Sinclair, 2004). For example, the concentration of leaf N presented a positive correlation with the photosynthetic rate and yield (Jin et al., 2011). In this sense, a canopy that allows maximum light interception as well as a sufficient N content in the leaves is essential for the conversion of radiation into biomass, and eventually, in production gain (Salvagiotti et al., 2008).

The maximum relative accumulation of N in the roots occurred at R_2_ (12%). This value is much lower than those reported by Unkovich et al. (2010) who found 33%, or 24% by Rochester et al. (1998) that was used by Salvagiotti et al. (2008) in an N balance calculation considering the contribution of roots.

In a review, Ciampitti and Salvagiotti (2018) studied the N balance in a wide dataset carried out from 1955 to 2017 in different locations around the world. Only in 17% of the observations the authors reported a positive N partial balance, with more than 80% of the data set pointing to negative N balances. Classifying the sites as low (0-42%), medium (42-72%) and high (72-98%) contribution of the BNF, they reached more clarifying results: In the first group, only 3% of the observations were positive, against 15% and 40% for the second and third groups, respectively, with mean balances of -100, -38.5 and -3.4 kg_[N]_ ha^-1^. The most limiting stressful conditions for BNF are high temperature and water deficit in terms of limiting rhizobia survival in the soil, nodulation, and N_2_ fixation (Hungria and Mendes, 2015).

Considering that in our observation the BNF contribution was in the middle range, the results of this study corroborate those obtained by these researchers. Without considering the root N partition, a review study reported that in 80% of cases, the partial N balance was negative with an average of -40 kg_[N]_ ha^-1^, but when the root N partition was considered, the balance was close to neutrality (-4 kg_[N]_ ha^-1^) (Salvagiotti et al., 2008). It is observed, therefore, that greater efficiency of BNF is necessary to seek positive N partial balances. Increased efficiency of BNF was associated with less negative balance, and 80% was necessary for neutrality (Santachiara et al., 2017). Also, in a study of N balance in temperate environments, positive values were reported with an average efficiency of BNF of 62% under an average yield of 4.0 Mg ha^-1^ and generated fewer N exports at harvest when compared to our data (Landriscini et al., 2018).

It is evidenced that as soybean yield increases, so does the N gap due to the higher demand for N in plants (Salvagiotti et al., 2008). Also, in environments with yields above 6,000 kg ha^-1^ and an average contribution of BNF around 67%, the N gap was 137 kg_[N]_ ha^-1^, as reported by Ciampitti and Salvagiotti (2018).

The facts described above show that in high yield environments (>6 Kg_[grains]_ ha^-1^) where the demand for N is greater, the N gap is greater even with a high contribution from BNF, suggesting the need to use other sources to supply N for plants. To reduce the N gap and supply the needed N for plants in such environments, it would primarily be through an effective symbiosis with highly adapted Rhizobium strains. For this, practices that contribute to good soil coverage are essential for maintaining a satisfactory temperature and water content condition for bacterial activity, in addition to correcting soil acidity and building a good soil profile for root exploration. Also, supplying plants with N at low rates, mainly during grain filling, aiming to mitigate the negative effect of NO_3_^-^ on the BNF process, can also be an alternative. The use of legumes in crop rotation schemes as well as the use of slow-release nitrogen fertilizers tend to be good options (Ciampitti and Salvagiotti, 2018). In a study of the influence of nitrogen fertilization on soybean yield, the application of slow-release urea below the root nodulation zone was efficient to increase grain yield, not inhibiting BNF activity (Salvagiotti et al., 2009).

The nitrogen harvest index (NHI) was 62.4% or 61.8% considering the N accumulated in the roots. In areas with an average yield of 3,480 kg ha^-1^, Bender et al. (2015) found an NHI of 73%. In a more productive environment (4,340 kg ha^-1^), an NHI of 86% was reported by Mastrodomenico and Purcell (2012). The high accumulation of N in the pods at R_8_ compared to the export by the grains suggests that a considerable part of the N was retained in the pod shells, a fact that may explain the lower Nitrogen harvest index (NHI) found in our study. On average, Mastrodomenico and Purcell (2012) concluded that 61% of the N accumulated by the crop is exported by the grains, and 32 kg_[N]_ Kg^-1^ of harvested grain remains in the crop residues. In our study, 34.5 kg_[N]_ Kg^-1^ was retained in the aerial crop residues, corroborating the results published by these researchers. Also, for a soybean crop with a yield of 4,850 kg ha^-1^, Salvagiotti et al. (2009) reported that 411 kg_[N]_ ha^-1^ were extracted by the plants, with 264 kg_[N]_ ha^-1^ being exported by the seeds and 147 kg_[N]_ ha^-1^ retained in the crop residues, generating a NHI of 64%. Thus, 30.3 kg_[N]_ ha^-1^ were accounted for in the residues per Kg of harvested grains (Salvagiotti et al., 2009), values close to those found in our study. It is important also to highlight that for a better determination of the N remobilization of leaf to the pod, as well as the quantification of the residual N of the senescent leaves fallen on the soil, it is strongly recommended to be collected and determine their total N.

## Conclusion

The characterization of N growth and accumulation in the studied soybean crop lead to a negative partial balance of N, even considering the contribution of N present in the roots, -33.4 kg_[N]_ ha^-1^. The N gap (the N from the soil) was 231.7 kg_[N]_ ha^-1^. It is recommended that when BNF is not enough to meet plant demand for N, generating a negative balance and a high N gap, to adopt techniques that increase the efficiency of BNF and the accumulation of N in the soil to balance these production systems in the medium to long terms.

Future research should focus on strategies to optimize the use of N by soybeans in order to reduce the N gap in the medium and long term. Among the strategies, an efficient use of N through an effective symbiosis with highly adapted Rhizobium strains is recommended, practices that contribute to good soil coverage and protection, in addition to correcting soil acidity and building a good soil profile for root exploration. Also, supplying plants with N at low rates, mainly during grain filling, aiming to mitigate the negative effect of NO_3_- on the BNF process, can also be an alternative.

## Data availability statement

The raw data supporting the conclusions of this article will be made available by the authors, without undue reservation.

## Author contributions

LZ, RU, and EB: methodology, data curation, and investigation. RU, FS, and KR: writing and editing. DD-N, RU, and KR: project administration, writing-editing, and review. JS and LI: investigation and data curation. LZ, FS, KR, and RU: conceptualization, validation, visualization, writing-editing, and review. All authors contributed to the article and approved the submitted version.
